# Addressing the serotonin hypothesis of depression through analyses of genetics, methylation and metabolite variations in glioma patients

**DOI:** 10.1038/s41598-025-25464-9

**Published:** 2025-10-28

**Authors:** Wendy Yi-Ying Wu, Beatrice Melin, Benny Björkblom, Rickard L. Sjöberg

**Affiliations:** 1 Department of Diagnostics and Intervention, Oncology, Umeå University, 90187 Umeå, Sweden; 2 Department of Chemistry, Umeå University, 90187 Umeå, Sweden; 3 Department of Clinical Science, Neurosciences, Umeå University, 90187 Umeå, Sweden

**Keywords:** Serotonin, MAOA, 5HTT, Glioma, Diseases, Genetics, Neurology, Neuroscience

## Abstract

Serotonin and serotonin metabolism has for decades been understood as playing a critical role in mood disorders and has more recently also been implicated in brain tumour biology. However, in part due to the lack of direct investigation of genetic and epigenetic variation affecting serotonin pathways within human brain tissue this understanding has recently been challenged. We analysed genetic and epigenetic variation in the *Monoamine oxidase A* (*MAOA)* and *serotonin transporter* (*5HTT)* genes using 232 biobanked glioma tissue samples from 216 adult patients. We further examined the association between use of antidepressants (targeting serotonergic pathways), serotonin levels and methylation. In male patients, genetic variation in the *MAOA* gene was significantly associated with tissue serotonin levels. Further analysis identified five single nucleotide variants (SNVs) that may contribute to this association. In contrast, *5HTT* variants were not statistically associated with serotonin pathway metabolites, nor were *MAOA* variants in females. Increased methylation at several *5HTT* CpG sites was positively correlated with serotonin levels and negatively correlated with 5-HIAA levels. In males, one CpG site in the *MAOA* gene was negatively associated with the 5-HIAA/serotonin ratio, suggesting reduced enzymatic degradation of serotonin due to lower *MAOA* activity. Patients using antidepressants had lower tissue serotonin levels. In males, genetic variation in the *MAOA* gene was significantly associated with tissue serotonin levels, although this association was not mediated by methylation. Our result supports the notion that the *MAOA* and *5HTT* genes are related to serotonin metabolism and that such metabolism is related to antidepressant use.

**Supplementary Information:**

The online version contains supplementary material available at 10.1038/s41598-025-25464-9.

## Introduction

**Fig. 1:**
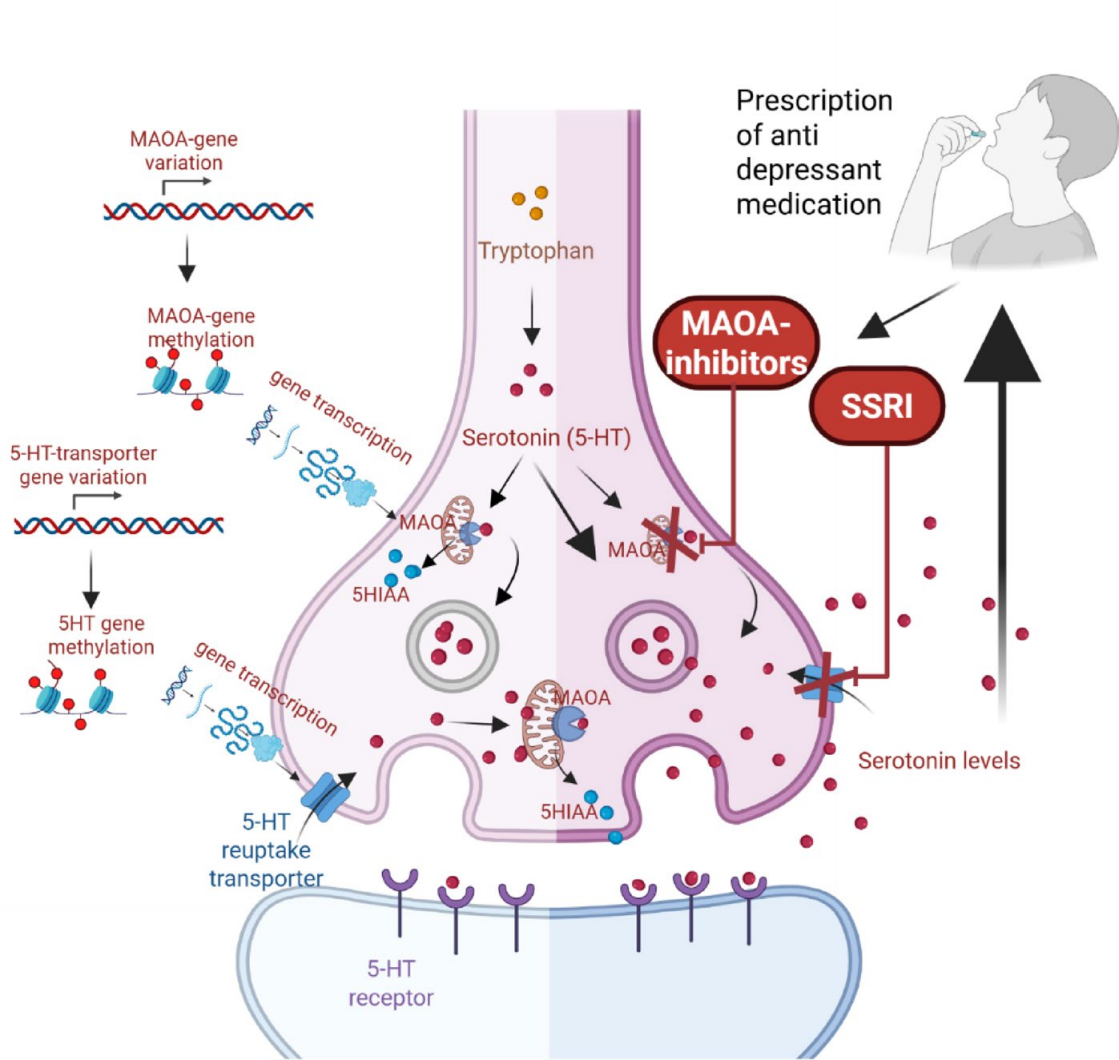
Overview of the serotonin system related to genetic and epigenetic regulation of *5HTT* and *MAOA* activity.

Use of antidepressant medication is common in the general population and even more common amongst patients with malignant gliomas^1^. The Monoamine Oxidase-A (*MAOA*) enzyme and the serotonin transporter (*5HTT also known as SCL6A4*) protein are both targets of pharmacological agents that have inspired much of the theoretical rationale for such treatments^2,3^. MAO-inhibitors were introduced as a remedy for depression in the 1950s and target the ability of a mitochondrial enzyme *MAOA* to contribute to the degradation of serotonin to its metabolic end-product 5-hydroxyindoleacetic acid (5-HIAA). The *5HTT*, which is the target of the serotonin re-uptake inhibitors, is a solute carrier protein that is responsible for removing serotonin (5HT) from the synaptic cleft back into the presynaptic neuron (Fig. 1). The effects of both compounds on psychiatric symptoms have been widely attributed to their presumed ability to regulate levels of monoamines in general and serotonin in particular in the central nervous system (CNS)^4–6^. This has in turn fostered the highly influential but also controversial understanding of untreated depression as reflecting serotonin deficiency^2–7^.

Approximately 40–50% of the variability in psychiatric disease, including depression, can be attributed to heritability^7–9^. Consistent with the serotonin hypothesis of depression, genetic variations in the X-linked *MAOA* gene *(*particularly in males who have one copy of the gene), and in the *5HTT* gene, located on chromosome 17, have been investigated as possible mechanisms contributing to heritability of depression for at least three decades^10,11^. The rationale is that genetic variations in these genes could (similar to the pharmacological agents discussed above) potentially affect serotonin levels in the CNS with subsequent effects on brain development, intermediate phenotypes and behavioural and psychiatric outcomes (Fig. 1)^12,13^.

However, the evidence for a stable and direct association between depression and serotonin levels remains inconclusive and contradictory^14,15^. Whereas the possible effects of *MAOA* and *5HTT* genes, for psychiatric disorders including depression has been studied for decades^10,11,16–18^ and evidence from knockout mouse and rat models support a link between *v*ariation in these genes and brain serotonin levels, direct evidence of such a relationship in humans remains limited^19–21^. Most available data are indirect and derived primarily from cerebrospinal fluid (CSF) measurements or neuroimaging, rather than from direct assessment in brain tissue^22,23^.

The present study represents an attempt to address these issues by using biobanked glioma tissue from 216 adult patients to investigate genetic and epigenetic regulation of serotonin metabolism focusing on the *5HTT* and *MAOA* genes. A further aim was to study the impact of serotonin levels on the individual prevalence of pharmacotherapy targeting this metabolism.

## Materials and methods

This study included 232 tumour tissue samples from 216 adult glioma patients diagnosed between year 2004 and 2016. Tumour tissue samples were obtained from patients undergoing neurosurgery at Umeå University Hospital. Prior to surgery, peripheral blood samples were collected for germline DNA analysis. Tumour tissue was collected during surgery and snap-frozen within 30–60 min. All samples were stored at −80 °C until further analysis^24^. Data on current medication use was recorded 1–4 days prior to the first surgery for all patients as part of the admission routine.

### Ethics approval and informed consent

The study was approved by the Swedish Ethical Review Authority (reference numbers 2023-03199-01, 2011/308 − 31 M, and 218/2003). All patients provided written informed consent. The study was conducted in accordance with Good Clinical Practice and the Declaration of Helsinki.

### Genotyping quality control, phasing and imputation

We reused existing genome-wide association study (GWAS) data from the Illumina OncoArray (*n* = 14) and Human 660 W (*n* = 31) platforms for this study. These datasets were originally generated for investigating germline variants associated with glioma risk (in the GICC and GLIOGENE consortium). These datasets were processed separately before merging with newly generated data from the OncoArray-500K_C platform (*n* = 163). Quality control (QC), phasing, and imputation were conducted uniformly across all datasets. After QC, a total of 203 subjects with validated genotyping data were included in the final analysis, with 5 subjects excluded from the OncoArray-500K_C dataset.

The following QC was applied: single nucleotide variants (SNVs) with a call rate of less than 0.98 and individuals with a call rate of less than 0.98 were excluded, and samples with sex discrepancies were removed. Given the *MAOA* gene’s location on the X chromosome, the dataset was split by sex for subsequent QC steps. In contrast, for variants located on chromosome 17, no splitting of the sample based on sex was performed. For SNV-level QC, we excluded variants with a minor allele frequency (MAF) of less than 0.01. Additionally, SNVs deviating from Hardy-Weinberg equilibrium (HWE *p* < 1e^−4^) were removed. We also excluded samples with a heterozygosity rate (FHAT) greater than 3 standard deviations from the mean and removed individuals with relatedness (pi-hat > 0.2) to retain only unrelated samples. After QC, phasing was performed using SHAPEIT4 and genotype imputation was conducted separately for each dataset using IMPUTE5 with the Haplotype Reference Consortium (Release 1.1) as reference panel. Based on genotyped data, we imputed variants in the *5HTT* gene, which encodes the serotonin transporter involved in serotonin reuptake, and the *MAOA* gene, which encodes the enzyme monoamine oxidase A, responsible for the degradation of serotonin.

### DNA methylation profiling

DNA methylation data were available for 225 of the 232 tumour tissues. In 7 tissues, profiling could not be performed due to insufficient remaining material for DNA extraction. Genome-wide DNA methylation profiling was performed using the Illumina Infinium MethylationEPIC BeadChip array (MethylationEPIC_v-1-0). Sample processing and hybridization were conducted according to the manufacturer’s protocol. The methylation data were analyzed using GenomeStudio software (version 2011.1; Illumina Inc.), with annotations based on the manifest file MethylationEPIC_v-1-0_B4.bpm and aligned to the human genome build GRCh37 (hg19).

To investigate the methylation status of the *MAOA* and *5HTT* genes, CpG sites were extracted based on the UCSC RefGene-based annotations. Probes were retained if the gene name field included either *MAOA* or *5HTT*, thereby capturing all CpG sites annotated within or near these genes. Beta values (representing the proportion of methylation at each CpG site, ranging from 0 to 1) were used to quantify methylation levels. In total, 31 CpG sites were identified for the *5HTT* gene and 24 CpGs for the *MAOA* gene. All included CpG sites passed standard quality threshold control (detection* P* values < 0.01).

### Metabolites

We included four metabolites involved in the serotonin pathway: tryptophan, serotonin, 5-HIAA, and kynurenine. Additionally, we calculated the ratio of 5-HIAA/serotonin to explore potential alterations in serotonin metabolism. These metabolites were measured as part of a previously published metabolomics dataset using gas chromatography-mass spectrometry (GC-MS) and liquid chromatography-mass spectrometry (LC-MS). To ensure comparability across samples, metabolite peak intensities were normalized using internal standards, accounting for potential variation introduced by sample handling, batch differences, or instrument fluctuations. Missing values attributed to biological factors or measurements below the detection limit were imputed using half the lowest observed value^24^.

### Statistical analysis

The Kruskal-Wallis test was used to assess the association between germline variants and metabolite levels. We applied the GATES (Gene-based Association Test using Extended Simes) method to combine* p* values of SNVs within each gene to obtain an overall gene-level* p* value that accounts for linkage disequilibrium among the SNVs^25^. Only genes with a gene-level* p* value < 0.05 were further investigated to avoid inflated type I error. Associations between methylation and metabolites were initially evaluated using Spearman correlation coefficients with results visualized in a heatmap. These associations were further examined using linear regression models to assess how changes in M-values (calculated as log_2_ [β / (1–β)]) related to metabolite levels (log_2_-transformed), adjusting for age, sex and antidepressant use. Multiple testing was controlled using the Benjamini-Hochberg false discovery rate (FDR) correction for the CpG–metabolite association tests, comprising 155 tests for the *5HTT* gene and 120 tests for the *MAOA* gene. Associations with FDR adjusted* p* value < 0.05 were considered statistically significant. Finally, methylation quantitative trait locus (meQTL) analyses were performed to investigate whether SNV identified in the metabolite-SNV association also influences methylation.

## Results

Supplementary Table 1 summarizes the characteristics of the study participants. The median age at diagnosis was 60 years (interquartile range 40–67) and 114 (53%) of the participants were male. Glioblastoma with IDH-wildtype was the most common subtype, accounting for 66% of the tumors. Other subtypes included astrocytoma (IDH-mutant: 7.8%; IDH-wildtype: 5.6%), glioblastoma IDH-mutant (3.9%), gliosarcoma (3.4%), and oligodendroglioma (IDH-mutant: 11%; IDH-wildtype: 2.6%). Fifteen patients had more than one surgery, including one who had three operations. The time interval between surgeries ranged from 0.15 to 5.13 years. Among the 216 glioma patients, the majority (89.8%) were not using antidepressant medication at the time of surgery. The most commonly used antidepressant was citalopram (6.5%), and other antidepressants included sertraline, mirtazapine, and escitalopram.

### SNV-metabolite associations

For the *5HTT* gene, a total of 538 SNVs were included, of which 210 SNVs were excluded due to low imputation quality (information score < 0.8), and 225 were non-polymorphic. Thus, 103 SNVs were retained for further analysis. For the *MAOA* gene, 816 SNVs were included, with 513 SNVs removed for low imputation quality (information score < 0.8), and 161 and 165 SNVs showed no polymorphism in males and females, respectively. Consequently, 142 SNVs (male) and 138 SNVs (female) were included for further analysis.

#### *5HTT* gene

The gene-level* p* value for the associations with tryptophan, serotonin, 5-HIAA, and kynurenine and ratio of 5-HIAA and serotonin were 0.703, 0.660, 0.576, 0.580, and 0.878, respectively. These results indicate no statistically significant association between *5HTT* and the levels of these metabolites.

#### *MAOA* gene in males

**Fig. 2:**
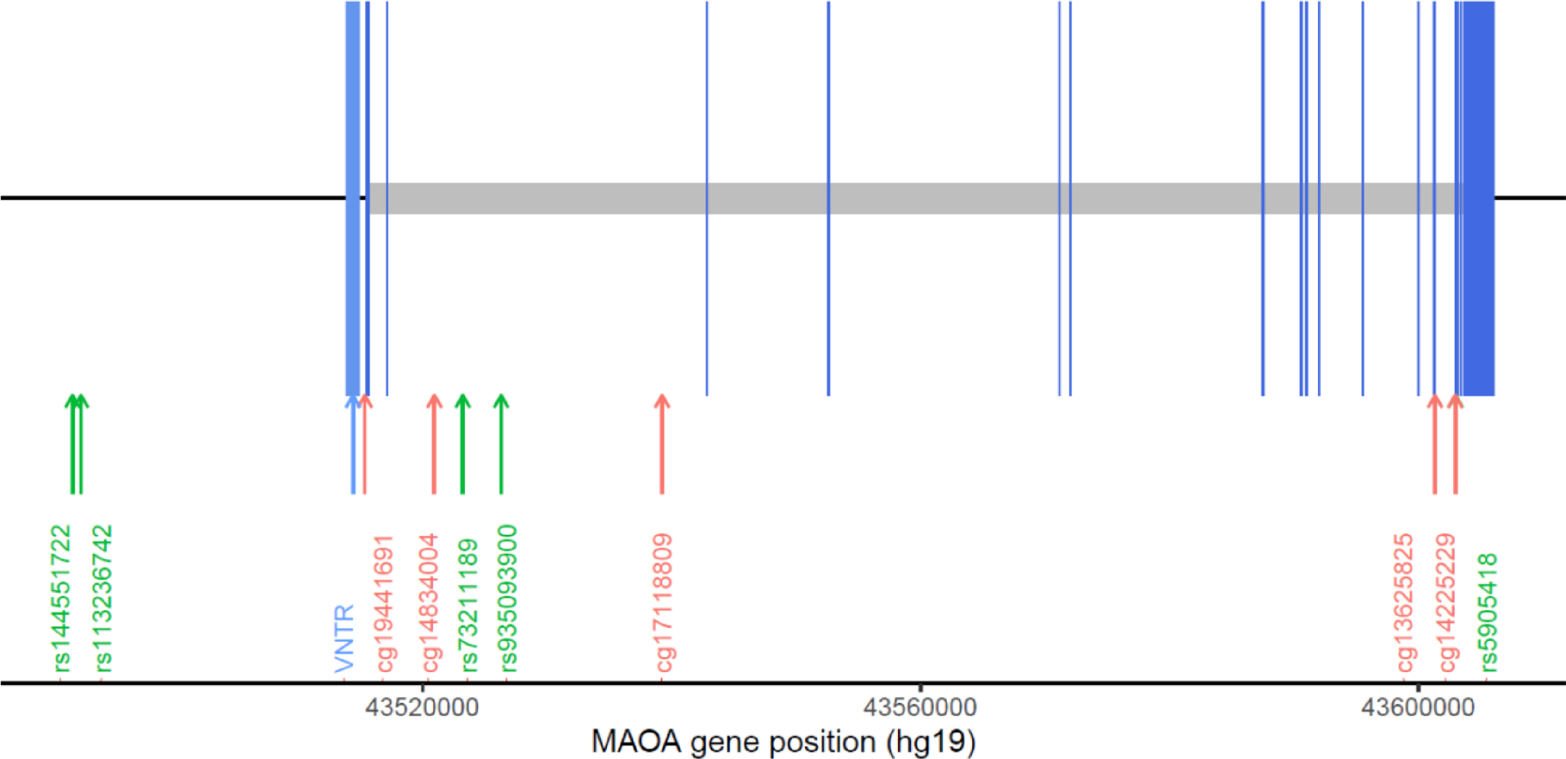
The gene structure of *MAOA* gene with associated single nucleotide variants (SNVs) and CpG sites. Arrows indicate the position of five SNVs associated with serotonin levels and CpG sites associated with serotonin pathway metabolites. CpG sites shown are significant at FDR-adjusted *p* < 0.05.

**Fig. 3:**
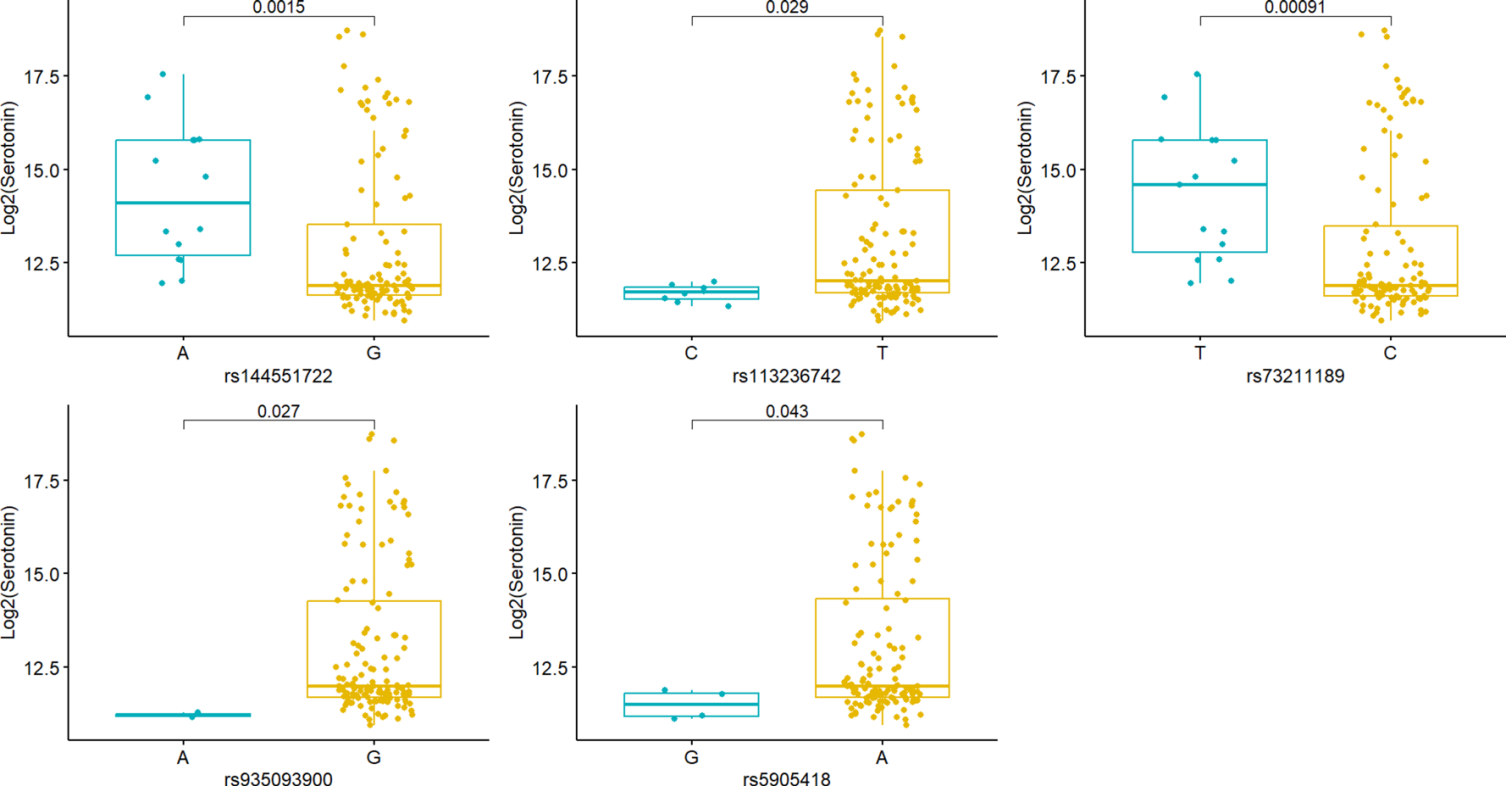
Boxplots of serotonin levels stratified by genotype for the five associated *MAOA* SNVs.

The gene-level* p* value for the association with tryptophan, serotonin, 5-HIAA, and kynurenine and ratio of 5-HIAA and serotonin were 0.371, 0.032, 0.322, 0.898, and 0.341, respectively. Only the association with serotonin reached statistical significance, suggesting a potential gene-level effect on serotonin levels. We further explored the variants that might be driving the gene-level association between *MAOA* and serotonin levels in tissue. Five SNVs (rs144551722, rs113236742, rs73211189, rs935093900, rs5905418) showed associations with serotonin levels (with* p* value < 0.05). Of these, rs144551722 and rs113236742 are located in intergenic regions, and the other three are intronic variants. The *MAOA* gene structure and the positions of these associated SNVs are illustrated in Fig. 2. Figure 3 presents serotonin concentrations stratified by genotype for these five SNVs.

#### *MAOA* gene in females

In contrast to the findings in males, no significant gene-level associations were observed with serotonin pathway metabolites (*p* values for tryptophan, serotonin, 5-HIAA, and kynurenine and ratio of 5-HIAA and serotonin were 0.666, 0.869, 0.690, 0.621, and 0.962, respectively).

### Methylation-metabolite associations

#### *5HTT* gene

In total, 31 CpG sites were identified in the *5HTT* gene. Distinct correlation patterns were observed between CpG methylation and tissue metabolites (Supplementary Fig. 1a). Increased methylation at several *5HTT* CpG sites was positively correlated with serotonin levels, while being negatively correlated with 5-HIAA levels. These opposing patterns suggest that DNA methylation at *5HTT* may influence serotonergic turnover or metabolism in the tissue. After correction for multiple testing,, 9 CpG sites were significantly associated with 5-HIAA, 4 with kynurenine and 9 with ratio of 5-HIAA and serotonin (FDR-adjusted* p* value < 0.05). Among the CpG sites associated with 5-HIAA, 6 were located in the gene body, 2 in the 5′ untranslated region (5′UTR), and 1 in the 3′UTR.

**Fig. 4:**
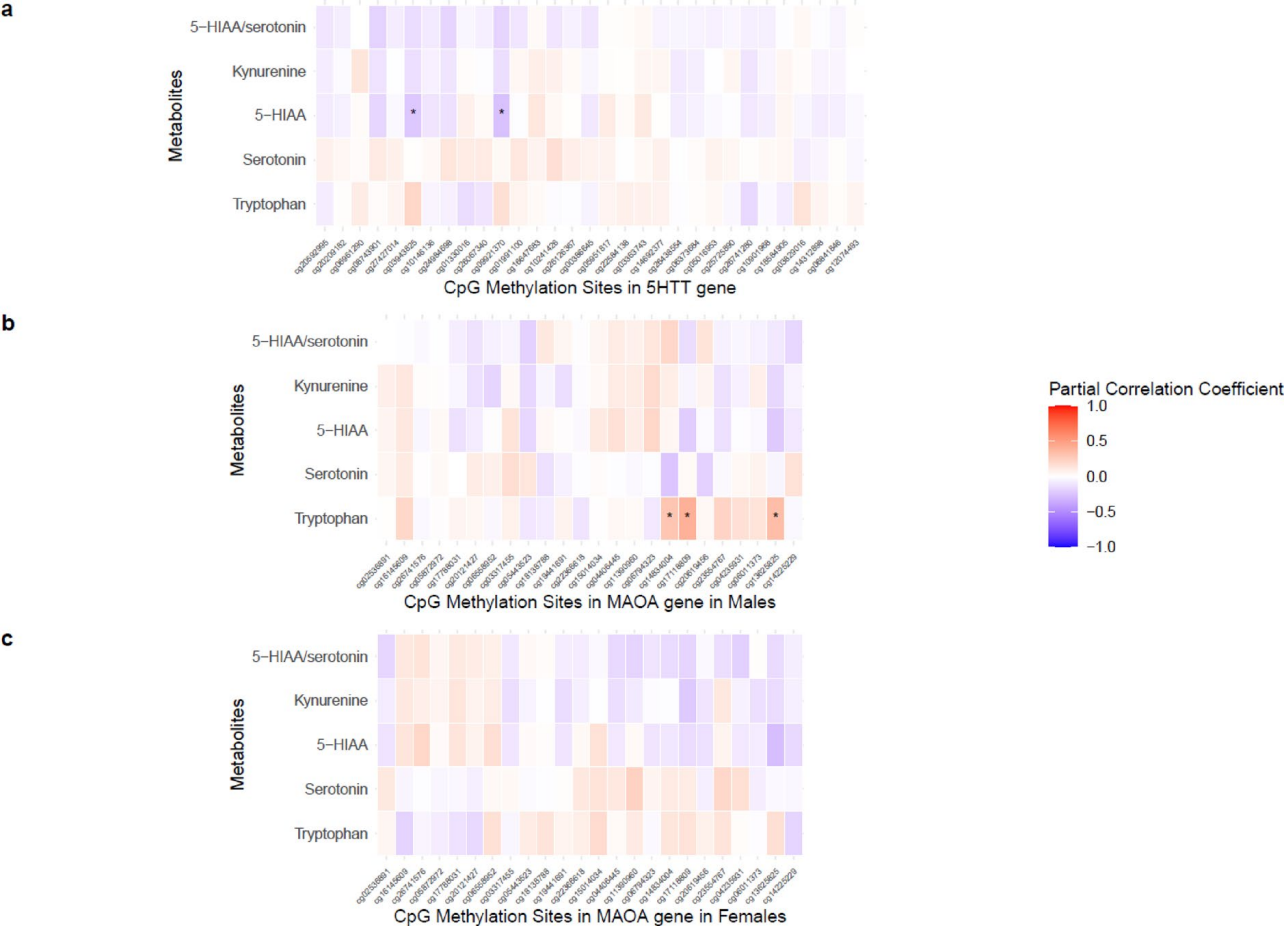
Partial correlation heatmaps of CpG methylation sites in *5HTT* (*n* = 31) and *MAOA* (*n* = 24) genes with metabolites, adjusted for age, sex (for *5HTT*) and anti-depressant use. An asterisk (*) indicates statistically significant associations after Benjamini-Hochberg false discovery rate correction at *p* < 0.05.

We further investigated the association between methylation M-values and metabolites using linear regression models adjusted for age, sex and antidepressant use. After Benjamini-Hochberg adjustment, only two CpG sites (cg09921370 and cg03943825) remained significantly associated with 5-HIAA (Fig. 4a, Supplementary Table 2). cg09921370 is located in the 5′UTR and cg03943825 is located within the gene body.

#### *MAOA* gene in males

For the *MAOA* gene in males, 3 CpG sites (cg14834004, cg17118809, and cg13625825) were significantly associated with tryptophan levels, 1 (cg14225229) with serotonin, 2 CpG sites with 5-HIAA, 1 with kynurenine and 1 with ratio of 5-HIAA and serotonin (Supplementary Fig. 1b). Among the CpG sites associated with 5-HIAA, cg17118809 was annotated as overlapping both the 5′UTR and gene body, and cg13625825 was located in the gene body. One CpG site (cg14225229) was negatively associated with the ratio of 5-HIAA and serotonin, indicating that higher methylation at these sites corresponds to a lower enzymatic activity involved in serotonin metabolism. However, after adjusting for age and antidepressant use, the association remained negative but did not reach statistical significance (FDR* p* value < 0.05). In contrast, three CpG sites associated with tryptophan remained significant after adjustment (Fig. 4b, Supplementary Table 3). We further investigated whether the SNV identified in the serotonin-SNV association also acts as a meQTL for the CpG site associated with serotonin levels. However, no significant associations were detected. This suggests that methylation does not mediate the observed genetic effects on serotonin levels, although limited statistical power due to small sample size may have contributed to the lack of detectable meQTLs.

#### *MAOA* gene in females

We didn’t observe significant associations between CpG sites and serotonin pathway metabolites in females, either before or after adjusting for age and antidepressant use (Fig. 4c, Supplementary Fig. 1c, Supplementary Table 4).

### Associations between antidepressant medication and serotonin pathway metabolites

**Fig. 5:**
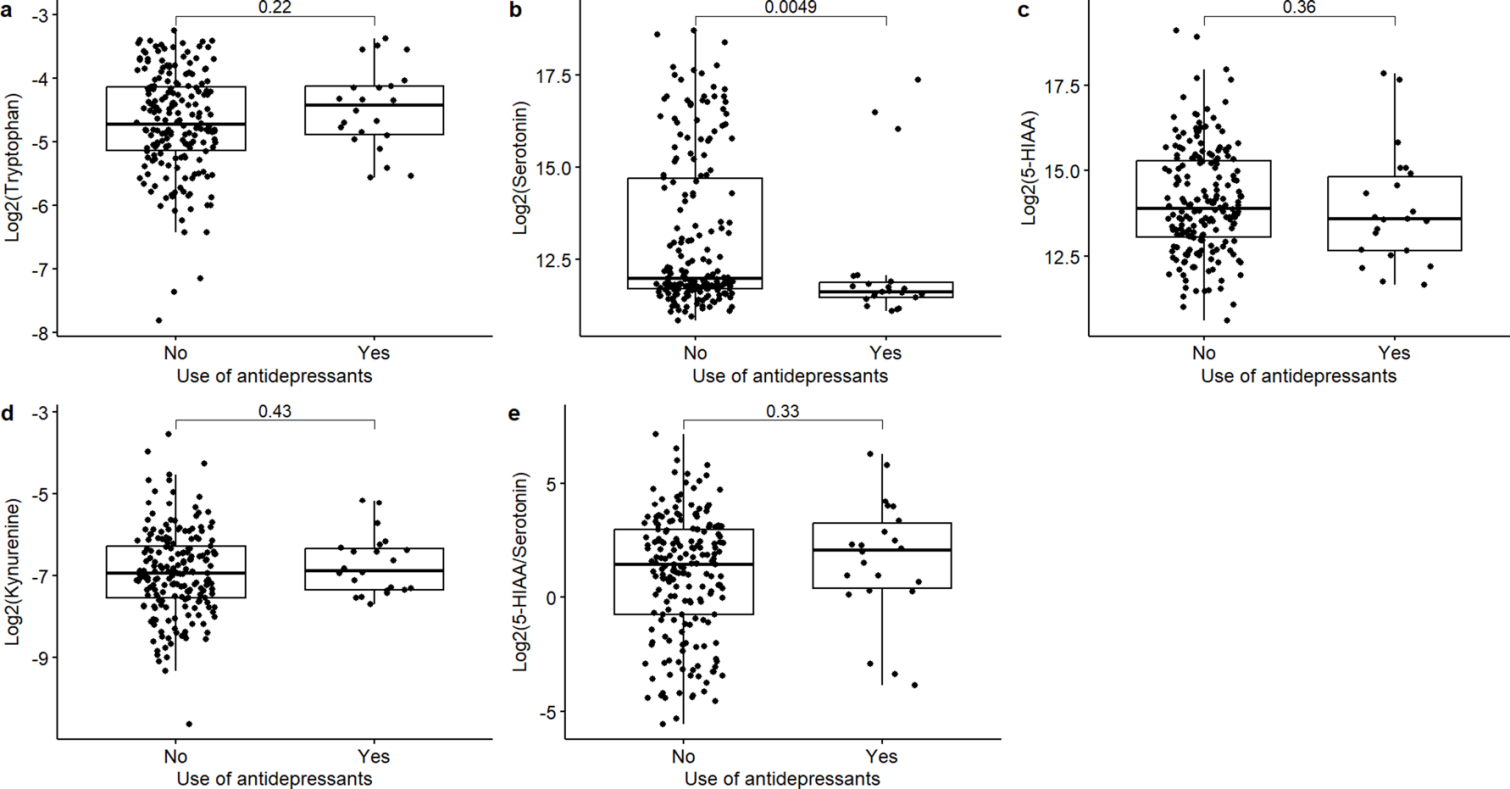
Tissue levels of serotonin pathway metabolites stratified by antidepressant use.

A significant association was observed between serotonin levels and use of antidepressant medication, with lower serotonin levels more commonly found in patients using antidepressant medication (Fig. 5, unadjusted* p* value = 0.005, FDR-adjusted* p* value = 0.025). No significant differences were observed in levels of tryptophan, kynurenine, 5-HIAA or the 5-HIAA/serotonin ratio between the two groups.

## Discussion

In this study, we investigated genetic and epigenetic variation in the *MAOA* and *5HITT* genes and their associations with serotonin pathway metabolites in glioma tissue. Our findings provide a uniquely detailed view of the influence of the relation of these much-studied genes serotonergic regulation in the brain tumour microenvironment. Notably, in male patients, genetic variation in *MAOA* was significantly associated with serotonin levels in tumour tissue and five SNVs were identified as potential contributors to this association. In contrast, no significant associations were found between *5HTT* variants and serotonin pathway metabolites, nor between *MAOA* variants and serotonin levels in females. DNA methylation at serotonergic genes showed regulatory potential, with higher methylation at *5HTT* CpG sites corresponding to elevated serotonin levels, and specific *MAOA* CpG sites exhibiting negative associations with the 5-HIAA/serotonin ratio, suggesting suppressed metabolic conversion of serotonin to 5-HIAA.

Building on these findings, several aspects warrant further discussion considering the existing literature. First, regarding genetic variation it should be noted that we did not directly test for the promoter polymorphisms that have been the focus of most of the genetic literature on the *MAOA* and *5HTT genes*. However, our genetic results were still broadly consistent with existing literature and metanalyses in which these regions have been directly covered. That is, we found significant effects of genetic variation in the *MAOA* gene in males on serotonin levels and serotonin metabolites^26,27^. Also consistent with this literature, results were less clear for women (where results are likely to be diluted by X-inactivation of one of the two alleles) and for the *5HTT* gene for both sexes^28^.

Finally, low levels of serotonin in glioma tissue were significantly associated with the use of antidepressant medication. The most reasonable interpretation for this finding seems to be that it mirrors a state of serotonin metabolism that is characteristic of patients that have been deemed to be in need of antidepressant treatment that the medication has been unable to compensate for. These results put the emerging epidemiological evidence suggesting a negative association between survival and use of antidepressants in patients with gliomas in perspective^1^. If this association is causal, it seems pharmacologically reasonable to assume that it mirrors low serotonin levels predicting use of antidepressants. This might support an interpretation of existing negative epidemiological associations between antidepressants and survival as confounded by depression per se rather than as caused by the pharmacological agent (which should work in the direction of increasing serotonin levels).

With regard to effects of methylation, provided that it (as is typically assumed) is associated with decreased gene activation, the directions of the effects in our study align with expected pharmacological agents targeting the proteins. Specifically, low activity (of the genes and subsequently the corresponding proteins) was in the present study associated with high serotonin levels. This highlights and may at first glance appear to be inconsistent with, the paradox that low function alleles are in the neurogenetic literature typically seen as the risk genes. Risk genes that, as illustrated by our findings, are likely to be associated with high serotonin levels rather than “serotonin deficiency”.

One possible resolution to this paradox could be that any risk conferred by such genotypes may be related to the role of serotonin levels during brain development and maturation. That is, genetically determined high serotonin levels during critical developmental stages may confer a risk for tilting cerebral development towards an intermediate phenotype that is vulnerable to conditions such as depression or aggression. This possibility could be reconciled with the notion that, in the adult individual, high serotonin levels may still be protective against such conditions^29,30^.

Our methylation data, when combined with metabolic data, also offer an interesting overview of the relation between *MAOA* and *5HTT* inhibition and the Serotonin/5-HIAA quotient. Here, it seems that gene inactivation (presumably associated with low function of the proteins), while associated with high serotonin levels, is also associated with low levels of 5-HIAA and by extension with a low 5-HIAA/serotonin ratio. This result also appears to be broadly consistent with molecular mechanisms. Inhibition of the *5HTT* is expected to reduce intracellular levels of serotonin by reduced reuptake, which would make it less available for degradation by the mitochondrial *MAOA* enzyme, resulting in higher serotonin and lower 5-HIAA. Likewise, direct inhibition of *MAOA* would be expected to produce a similar effect.

Finally, there is also a neuro-oncological dimension to our findings, in that it adds interesting perspectives to the circumstantial evidence suggesting that there might be a link between serotonin metabolism, antidepressant medications, and gliomas^1,31–34^. Of particular interest in the present context was the SNV rs144551722 in the *MAOA* gene, that we have previously shown to be associated with the development of glioblastomas in males in two large independent samples^34^. The risk variant of this SNV (G) was in the present study significantly associated with low serotonin levels in glioma tissue from male patients as compared with the less common A variant. This finding, which does seem to further validate the identification of an effect of this gene variation, could tentatively suggest that low serotonin levels might exert a slight protective effect against glioma development. This could be interpreted as consistent with the finding that use of antidepressants, as shown in a recent meta-analysis, seems to have a small protective effect against the development of gliomas, provided that such use is seen as a marker for low serotonin levels^31^. However, further studies would be necessary before any firm conclusions are drawn.

The present study of serotonin metabolism in glioma patients represents an important advance in relation to previous literature, in that it illuminates more parts of a molecular chains of events, that have until now remained largely hidden in the clinical literature based on human subjects. That is, much of our current understanding of the molecular mechanisms of depression as it plays out in brain tissue of otherwise normal humans, as well as in glioma patients is based on assumptions about events related to serotonin metabolism^6,14,16^. These assumptions have covered chains of events from genotype and methylation to metabolism and behaviour. In this study, we demonstrated that significant step by step relations between all of the studied layers of events in the serotonin metabolism can indeed be found. For instance, our results suggest both that there are significant links between the *5HTT* and *MAOA* gene variation and methylation, and serotonin levels, as well as between serotonin levels and the choice to accept antidepressant medication. However, these results were not always strong and clear-cut with a number of negative findings that may seem surprising from the perspective of the existing literature.

Several limitations should be considered when interpreting our findings. First, the study was conducted using glioma tissue, which may not fully reflect methylation and metabolite patterns in normal brain tissue. Further work is needed to validate the relationships in individuals without neurological disorders, and broad generalizations to healthy individuals should be made with caution. Second, as an observational study, the findings are correlational and do not establish causality. For example, antidepressant use was recorded 1–4 days prior to the first surgery, so temporal relationships cannot be fully determined. Third, some relevant environmental factors, such as duration of antidepressant use or other substance use, were not available in the dataset, limiting our ability to fully account for their effects. Finally, a formal power analysis was not performed prior to the study. Although we detected significant associations, the sample size may have limited the ability to identify smaller effects.

In sum, the present study demonstrates that the epigenetic and genetic regulation of serotonin metabolism as well as its relation to antidepressant treatment is complicated. But still, our results seem to broadly support the notion that genetic and epigenetic variation in the *MAOA*, and *5HTT* genes influence serotonin metabolism and that serotonin metabolism affects depression. The results also suggest that further exploring the relation between serotonin metabolism, depression, and incidence of and survival in gliomas might be a worthwhile effort.

## Supplementary Information

Below is the link to the electronic supplementary material.

Supplementary Material 1

Supplementary Material 2

## Data Availability

The datasets used and analysed during the current study available from the corresponding author on reasonable request.
