# Supplementary material for: Addressing the serotonin hypothesis of depression through analyses of genetics, methylation and metabolite variations in glioma patients
Source: Sci Rep. 2025 Oct 28;15:37732. doi: 10.1038/s41598-025-25464-9 (PMC12569211; doi:10.1038/s41598-025-25464-9)

Supplementary Figure 1. Spearman correlation heatmaps of CpG methylation sites in *5HTT*(n=31) and *MAOA*(n=24) gene with metabolites. An asterisk (*) indicates statistically significant associations after Benjamini-Hochberg false discovery rate correction at *P* < 0.05.


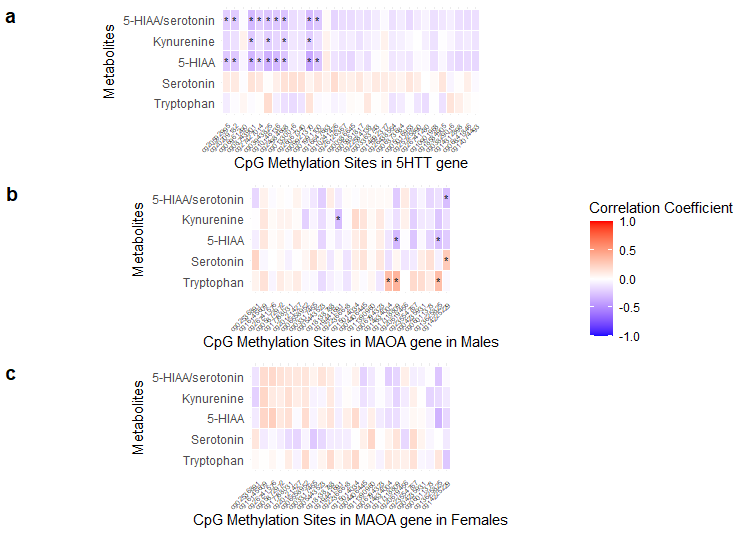

Supplement: Supplementary file 1 — Supplementary Material 1 [file 41598_2025_25464_MOESM1_ESM.docx]
